# Cell cycle profile data on splenocytes of high fat diet induced obese mice treated with ferulic acid

**DOI:** 10.1016/j.dib.2020.105990

**Published:** 2020-07-05

**Authors:** Hyuna Lee, Jin Ah Cho, Eunmi Park

**Affiliations:** a Department of Food and Nutrition, Hannam University, 70, Hannam-ro, Daedeok-gu, Daejeon 306-791, Republic of Korea; b Department of Food and Nutrition, Chungnam National University, 99, Daehak-ro, Yuseong-gu, Daejeon 34134, Republic of Korea

**Keywords:** Ferulic acid, Obesity, Splenocytes, Cell cycle, Flow cytometry

## Abstract

The reported data are related to the article entitled “Ferulic acid maintains the self-renewal capacity of embryo stem cells and adipose-derived mesenchymal stem cells in high fat diet-induced obese mice” [1]. Ferulic acid is a natural bioactive compound and demonstrated potential to serve as a self-renewing biomarker in an alkaline phosphate assay and caused increased Nanog mRNA levels in embryonic stem cells. In these data, we examined another functional aspect of ferulic acid, namely the effect of ferulic acid on the cell cycle of splenocytes.

These data were collected from the splenocytes of C57BL/6 J male mice that were fed either a high fat diet (HFD) alone or an HFD diet supplemented with ferulic acid (5 g/kg diet) for 8 weeks. As expected, the HFD resulted in a significant increase in mouse body weight, liver weight, and epididymal fat tissue weight compared to the control diet (Cho and Park, 2020). The cell cycle profile of mouse splenocytes in HFD-induced obese mice was evaluated by FACS. Since the G1 checkpoint is the point at which cells enter the cell cycle, an internal or external stimulation can cause the cell to delay passing G1 and instead enter a quiescent state known as G0 without proceeding past the restriction checkpoint. DNA damage is the main trigger that can cause a cell to "restrict" itself and not enter the cell cycle [2]. These results show that ferulic acid helps attenuate G1/S arrest in splenocytes in HFD-induced obese mice.

Specifications table

**Table:** 

Subject	Biology
Specific subject area	Obesity and Cell Metabolism
Type of data	Table
	Figure
How data were acquired	The cell cycle profile of splenocytes with and without ferulic acid treatment in high fat diet-induced obese mice were acquired by fluorescence-activated cell sorting (FACS) using a Beckman system with analysis software.
Data format	Raw and analysed
Parameters for data collection	Mice were fed on a 60% high fat diet with a 5 g/kg ferulic acid supplement for 8 weeks (*n* = 5 per group) and splenocytes were collected for FACS analysis.
Description of data collection	The effect of ferulic acid on the cell cycle profile of splenocytes from high fat diet-induced obese mice were acquired by fluorescence-activated cell sorting (FACS) analysis.
Data source location	Hannam University, Daejeon, Republic of Korea
Data accessibility	Data is accessible with this article and provided in the supplementary material.
Related research article	Cho, J., Park, E. Ferulic acid maintains the self-renewal capacity of embryo stem cells and adipose-derived mesenchymal stem cells in high fat diet induced obese mice Journal of Nutritional Biochemistry doi:10.1016/j.jnutbio.2019.108327

## Value of the data

• These data show the cell cycle phenotype of splenocytes in HFD-induced obese mice.
• These data can be used by scientists studying the effect of ferulic acid on immunological and cell cycle-related analysis, which can be further developed based on these data.
• These data give insights into the link between metabolic syndrome and immune-related diseases.

## 1 Data description

**Fig. 1:**
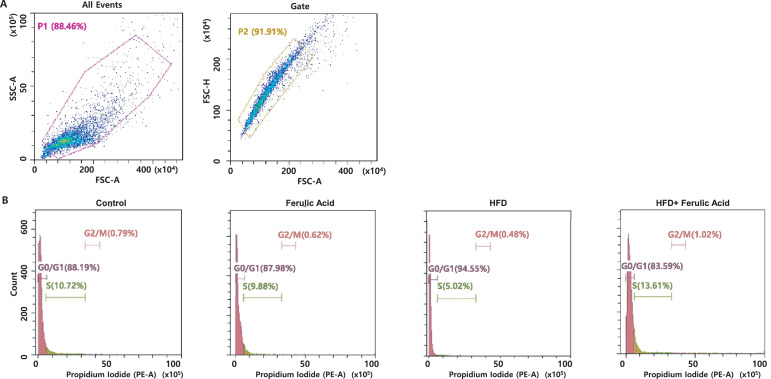
**Ferulic acid reduces the G0/G1 cycle population in splenocytes of HFD-induced obese mice**. Splenocytes were obtained from mice and used for FACS analysis and representative plots show the side scatter (SS) and forward scatter (FS) in all events (A) to identify single cells. The splenocytes were cultured with 5μg/ml Con A for 48 h, fixed with 70% ethanol, stained with PI, and then analysed by FACS (B). Control, standard chow diet; Ferulic acid, standard chow diet with ferulic acid (5 g/kg diet); HFD, high fat diet; HFD + Ferulic acid, high fat diet with ferulic acid. The parameters for FACS analysis are referred to as SSC-A: Side Scatter-A; FSC-A: Forward Scatter-Area; and FSC—H: Forward Scatter-Height.

The cell cycle profile of mouse splenocytes in HFD-induced obese mice was evaluated. The data show the representative cell cycle profile (Fig. 1) and the percentages of the cell population in different cell cycle phases are summarized in Table 1 (raw data is in Supplementary Material).

**Table 1:** Percentage of cell counts in different phase of cell cycle.

Group	Percentage of cell count in different phases (%)		
	G0/G1	S	G2/M
**Control**	89.9 ± 3.8^a^	7.9 ± 2.6	0.8 ± 0.2
**Ferulic acid**	89.1 ± 11.2^a^	9.4 ± 9.5	0.6 ± 0.5
**HFD**	94.3 ± 3.4^b^	5.0 ± 2.7	0.3 ± 0.3
**Ferulic acid + HFD**	84.1 ± 13.2a	14.1 ± 1.6	0.7 ± 0.3

All data were expressed as mean±SEM and analyzed two-way ANOVA with Tukey's post hoc test. Superscripts with different letters indicate significant differences between the groups. Control, standard chow diet; Ferulic acid, standard chow diet with ferulic acid (5 g/kg diet); HFD, 60% of high fat diet; HFD + Ferulic acid, 60% of high fat diet with ferulic acid.

Table 1 showed that there was no statistical difference in cell populations in the G0/G1 cycle between the control group, ferulic acid group, and ferulic acid with HFD group. In marked contrast, the HFD group showed a significant increase in the G0/G1 cycle population. Splenocytes in HFD-induced obese mice (94.3%) stay in the G0/G1 phase of the cell cycle significantly longer than splenocytes of mice fed a standard chow diet (control, 89.9%, *P*<0.05), ferulic acid supplementation (89.1%, *P*<0.05), or HFD with ferulic acid supplementation (84.1%, *P*<0.05). Interestingly, ferulic acid significantly reduced the length of the G0/G1 phase of the cell cycle of splenocytes in HFD-induced obese mice compared to the HFD group (84.1% versus 94.3%). In addition, there was no statistical difference in the cell populations in either the S cycle or the G2/M cycle between the four groups. Therefore, these results show that ferulic acid helps attenuate G1/S arrest in splenocytes from HFD-induced obese mice.

## 2 Experimental design, materials, and methods

### 2.1 Mouse experiment

Six-week old male C57BL/6 mice were housed in groups of five per cage with food and tap water ad libitum in a pathogen-free animal care facility. The facility environment had a 12:12 h light–dark cycle, 50±5% humidity, and a temperature of 20±1 °C. The mice were fed either a high fat diet (60 kcal% fat, D12492, Research diet, USA) or a standard chow control diet (10 kcal% fat, D12492 diet Match 7% Sucrose, Research diet, USA) with ferulic acid supplement (5 g/kg diet) for 8 weeks. The groups were divided as follows: mice fed with a standard chow diet (Control), mice supplemented with ferulic acid (Ferulic acid), mice fed with HFD (HFD), and mice fed with HFD and supplemented with ferulic acid (HFD+Ferulic acid). Each group contained five mice. All animal procedures and care were conducted using the approved animal protocol as per the guidelines of the IACUC.

### 2.2 Flow cytometry

After 8 weeks, C57/BL6 male mice were sacrificed, and mouse spleens were isolated for ex vivo splenocytes culture, as described in Ref. [1]. For flow cytometry analysis, the mouse splenocytes were minced and isolated using a cell strainer with 45 μm diameter pores and washed twice with PBS. The splenocytes were cultured in RPMI media and stimulated with Concanavalin A (5 μg/ml) for 48 h, fixed with 70% ethanol, stained with propidium iodide (PI), and then analysed by fluorescence-activated cell sorting (FACS) on a Beckman system using FACS analysis software.

### 2.3 Statistical analysis

Data shown are reported as the mean ± standard error (SE) and statistical analysis was conducted using two-way repeated measures ANOVA followed by a Tukey's post hoc test.

## Declaration of Competing Interest

The authors declare that they have no known competing financial interests or personal relationships which have, or could be perceived to have, influenced the work reported in this article.

## Appendix Supplementary materials

Image, application 1
